# Post-transplant cyclophosphamide versus antithymocyte globulin in patients with acute myeloid leukemia in first complete remission undergoing allogeneic stem cell transplantation from 10/10 HLA-matched unrelated donors

**DOI:** 10.1186/s13045-020-00923-0

**Published:** 2020-07-03

**Authors:** Eolia Brissot, Myriam Labopin, Ian Moiseev, J. J. Cornelissen, Ellen Meijer, Gwendolyn Van Gorkom, Montserrat Rovira, Fabio Ciceri, Laimonas Griskevicius, Didier Blaise, Edouard Forcade, Martin Mistrik, Stephan Mielke, Claude Eric Bulabois, Riitta Niittyvuopio, Eric Deconinck, Annalisa Ruggeri, Jaime Sanz, Alexandros Spyridonidis, Bipin Savani, Sebastian Giebel, Arnon Nagler, Mohamad Mohty

**Affiliations:** 1 Sorbonne Université, AP-HP, INSERM UMRs938, Paris, France; 2 Service d’Hématologie clinique et de Thérapie cellulaire, Assistance Publique-Hôpitaux de Paris, Hôpital Saint-Antoine, 184, rue du Faubourg Saint Antoine, 75012 Paris, France; 3 European Society for Blood and Marrow Transplantation Paris Study Office/CEREST-TC, Paris, France; 4 R.M. Gorbacheva Memorial Institute of Oncology, Hematology and Transplantation, Pavlov First Saint Petersburg State Medical University, St. Petersburg, Russian Federation; 5 Department of Hematology, Erasmus University Medical Center, Rotterdam, the Netherlands; 6 Amsterdam University Medical Center, VU Medical Center, Department of Hematology, Cancer Center Amsterdam, Amsterdam, Netherlands; 7 Dept. Internal Medicine, Hematology/Oncology, University Hospital Maastricht, Maastricht, The Netherlands; 8 Apheresis & Cellular Therapy Unit, Department of Hemotherapy and Hemostasis, ICMHO, Hospital Clínic, University of Barcelona, Barcelona, Spain; 9 Hematology and Bone Marrow Transplantation, IRCCS San Raffaele Scientific Institute, Milan, Italy; 10 University Vita-Salute San Raffaele, Milan, Italy; 11 Hematology, Oncology & Transfusion Center, Vilnius University Hospital Santaros Klinikos, Vilnius University, Vilnius, Lithuania; 12 Centre de Recherche en Cancérologie de Marseille, Institut Paoli Calmettes, Marseille, France; 13 CHU Bordeaux, Hospital Haut-Leveque, Pessac, France; 14 Department of Haematology and Transfusion Medicine, University Hospital and Comenius University, Bratislava, Slovak Republic; 15 Department of Internal Medicine II, University Hospital of Würzburg, Oberdürrbacher Str. 6, D-97080 Würzburg, Germany; 16 Department of Hematology, CHU de Grenoble, Grenoble, France; 17 HUCH Comprehensive Cancer Center, Stem Cell Transplantation Unit, Helsinki, Finland; 18 Service d’Hématologie, Hopital Jean Minjoz, Besançon, France; 19 Department of Haematology, University Hospital La Fe, University of Valencia, Valencia, Spain; 20 Department of Haematology, Centro de Investigación Biomédica en Red de Cáncer, Instituto Carlos III, Madrid, Spain; 21 Department of Internal Medicine, Bone Marrow Transplantation Unit, University Hospital of Patras, Patras, Greece; 22 Long Term Transplant Clinic, Vanderbilt University Medical Center, Nashville, TN USA; 23 Department of Bone Marrow Transplantation and Oncohematology, Maria Sklodowska-Curie Memorial Cencer Center and Institute of Oncology, Gliwice, Poland; 24 Hematology Division, BMT and Cord Blood Bank, Chaim Sheba Medical Center, Tel-Hashomer, Israel

**Keywords:** Post-transplant cyclophosphamide, Antithymocyte globulin, Matched unrelated donor, Acute myeloid leukemia

## Abstract

**Background:**

Graft-versus-host disease (GVHD) remains a major contributor to mortality and morbidity after allogeneic stem-cell transplantation (allo-HSCT). The updated recommendations suggest that rabbit antithymocyte globulin or anti-T-lymphocyte globulin (ATG) should be used for GVHD prophylaxis in patients undergoing matched-unrelated donor (MUD) allo-HSCT. More recently, using post-transplant cyclophosphamide (PTCY) in the haploidentical setting has resulted in low incidences of both acute (aGVHD) and chronic GVHD (cGVHD). Therefore, the aim of our study was to compare GVHD prophylaxis using either PTCY or ATG in patients with acute myeloid leukemia (AML) who underwent allo-HSCT in first remission (CR1) from a 10/10 HLA-MUD.

**Methods:**

Overall, 174 and 1452 patients from the EBMT registry receiving PTCY and ATG were included. Cumulative incidence of aGVHD and cGVHD, leukemia-free survival, overall survival, non-relapse mortality, cumulative incidence of relapse, and refined GVHD-free, relapse-free survival were compared between the 2 groups. Propensity score matching was also performed in order to confirm the results of the main analysis

**Results:**

No statistical difference between the PTCY and ATG groups was observed for the incidence of grade II–IV aGVHD. The same held true for the incidence of cGVHD and for extensive cGVHD. In univariate and multivariate analyses, no statistical differences were observed for all other transplant outcomes. These results were also confirmed using matched-pair analysis.

**Conclusion:**

These results highlight that, in the10/10 HLA-MUD setting, the use of PTCY for GVHD prophylaxis may provide similar outcomes to those obtained with ATG in patients with AML in CR1.

## Background

Graft-versus-host disease (GVHD) remains a major contributor to mortality and morbidity after allogeneic stem-cell transplantation (allo-HSCT) [1–3]. The pathogenesis of acute GVHD (aGVHD) and chronic GVHD (cGVHD) is complex [4, 5]. Acute GVHD is initiated when alloreactive donor immune cells recognize immunologically disparate antigens in the host. The risk of developing GVHD depends on the degree of HLA match, recipient age, graft source, underlying disease diagnosis, intensity of conditioning regimen, and also on GVHD prophylaxis. The updated recommendations suggest that rabbit antithymocyte globulin or anti-T-lymphocyte globulin (ATG) should be used for GVHD prophylaxis in patients undergoing matched-unrelated donor (MUD) allo-HSCT [6]. This recommendation is based on several high-level evidence publications showing a decreased rate of both acute and chronic GVHD [7–10]. However, ATG delays immune reconstitution and is associated with more infections, especially viral [11–13]. On the other hand, post-transplant cyclophosphamide (PTCY) is now well-established, successful, and widely utilized for GVHD prophylaxis after haploidentical allo-HSCT [14, 15]. The mechanism of PTCY has been described as inducing preferential elimination and clonal deletion of alloreactive T cells [16, 17]. Moreover, there is evidence supporting regulatory T cell importance in mediating long-term post-transplant tolerance and GVHD control with PTCY [18–20]. Since then, PTCY has been applied in other settings, including HLA-identical sibling or UD and mismatched unrelated donor (MMUD) [21–24]. In the 9/10 MMUD setting, PTCY use was described as effective anti-GVHD prophylaxis compared to ATG and likely to provide better outcomes in long-term disease control [25]. This increase in evidence of the positive impact of PTCY, prompted us to evaluate its practical clinical use in allo-HSCT with MUD.

In the current study, we retrospectively analyzed results of allo-HSCT transplantation using 10/10 MUD in a homogenous population of acute myeloid leukemia (AML) patients in first complete remission (CR1), comparing the outcomes of PTCY versus ATG as GVHD prophylaxis.

## Methods

This is a retrospective study from the Acute Leukemia Working Party (ALWP) of the European Society for Blood and Marrow Transplantation (EBMT), which is a working group of more than 600 transplant centers, mostly located in Europe, that are required to report annually all consecutive transplantations and follow-up data. Data from all EBMT centers are entered, managed, and maintained in a central online database. There are no restrictions on centers for reporting data, except for those required by law on patient consent, data confidentiality, and accuracy. Quality control measures include several independent systems: confirmation of the validity of the entered data by the reporting team, selective comparison of the survey data with MED-A data sets in the EBMT registry database, cross-checking with the National Registries, and regular in-house and external data audits. Patients provide informed consent authorizing the use of their personal information for research purposes. Each patient provides consent for transplant according to the ethical principles of the Declaration of Helsinki. The study was approved by the Institutional Review Board of the ALWP of the EBMT.

### Eligibility criteria

In order to be included in this study, patients had to fulfill all of the following criteria: age ≥ 18 years; diagnosed with AML and undergoing first HSCT in CR1; from a 10/10 MUD (patients and donors should have HLA A, B, C, and DRB1 and DQB1 allelic typing performed). Graft source of stem cells was the peripheral blood stem cells (PBSC) or bone marrow (BM). In the ATG group, allo-HSCT patients received 5 mg/kg of thymoglobulin. All patients underwent transplantation between January 2010 and December 2017.

### Definitions

Endpoints of the study were the cumulative incidence of acute GVHD grade II–IV and chronic GVHD, leukemia-free survival (LFS), overall survival (OS), refined GVHD-free, relapse-free survival (GRFS), cumulative incidences of relapse (RI), and non-relapse mortality (NRM). Acute GVHD was graded according to the modified Glucksberg criteria [26] and cGVHD according to the revised Seattle criteria [27].

Engraftment was defined as achieving an absolute neutrophil count greater than or equal to 0.5 × 10^9^/L for three consecutive days. The probability of being alive without evidence of relapse or progression defined LFS. OS was defined as the time from allo-HSCT to death, regardless of the cause. Refined GRFS was defined as being alive with neither grade III–IV aGVHD nor severe cGVHD nor disease relapse at any time point ^15^. Death without evidence of relapse defined NRM [28].

The cytogenetic risk was defined according to the MRC criteria ^11^. Performance status was graded according to the Karnofsky Performance Status (KPS) scale and was defined as poor when it was < 90%. The conditioning regimen was defined according to data reported by the EBMT centers as myeloablative (MAC) or reduced-intensity (RIC) according to the EBMT definition ^12^.

### Statistical analysis

Median values, inter-quartiles ranges (IQR), and minimum and maximum were used to express continuous variables while frequencies and percentages were used for categorical variables. Patient-, disease-, and transplant-related variables of the groups were compared using the chi-square or Fischer’s exact test for categorical variables, and the Mann-Whitney test for continuous variables [29].

Acute and chronic GVHD, RI, and NRM were calculated using the cumulative incidence estimator to accommodate competing risks. For NRM, relapse was the competing risk, and for RI, the competing risk was death without relapse. To study acute and chronic GVHD, we considered relapse and death to be competing events. The probabilities of OS, LFS, and GRFS were calculated using the Kaplan–Meier method. Univariate analyses were done using Gray’s test for cumulative incidence functions and the log-rank test for OS, GRFS, and LFS. A Cox proportional hazards model was used for multivariate regression. All variables differing significantly between the two groups, or variables deemed conceptually important were included in the Cox model: ATG versus PTCY, age, year of transplant, time from diagnosis to transplant, secondary versus de novo AML, cytogenetics, KPS, conditioning regimen, female donor to male recipient versus other, stem cell source, CMV serology status for both patients, and donors. In order to test for a center effect, we introduced a random effect or frailty for each center into the model [30, 31]. Results were expressed as the hazard ratio (HR) with the 95% confidence interval (95% CI). *P* values were two-sided. Propensity score matching was also performed in order to confirm the results of the main analysis ^19^. Each patient identified as having received PTCY was matched with two patients who had received ATG. The following factors were included in the propensity score model: age, time from diagnosis to transplant, secondary AML, cytogenetics, conditioning intensity, female donor to male recipient, patient, and donor CMV serology status. All tests were two-sided. The type I error rate was fixed at 0.05 for the determination of factors associated with time-to-event outcomes. Statistical analyses were performed with the SPSS 24 (SPSS Inc./IBM, Armonk, NY, USA) and R 3.6.2 (R Development Core Team, Vienna, Austria) software packages.

## Results

The baseline characteristics are summarized in Table 1. Overall, 174 and 1452 patients receiving PTCY and ATG, respectively, fulfilled the inclusion criteria. The median follow-up period was 20.5 (IQR 6.9–32.6) months in the PTCY group as compared to 33.2 (17.6–52.7) months in the ATG group (*p* < 0.001). Patients in the PTCY group were younger (median age 46 versus 56 years, *p* < 0.001) and had undergone allo-HSCT more recently (median year of allo-HSCT 2016 versus 2014, *p* < 0.001). Time from diagnosis to allo-HSCT was similar in both groups. Peripheral blood stem cells were the more frequently used stem cell source, with no significant difference of utilization among the 2 groups. Conditioning regimen intensity was comparable in the two groups. The only ATG brand used was Thymoglobuline®. Two or three additional immunosuppressive agents were used in 55% and 85% of patients receiving PTCY and ATG, respectively (supplementary data, Table S1).

**Table 1:** Baseline characteristics of patients

*N*	ATG	PTCY	Test *p* value
	1452	174	
**Follow-up**			
Median time (IQR) mo	33.2 (17.6–52.7)	20.5 (6.9–32.6)	< 0.001
**Age at allo-HSCT**			
Median (range) [IQR]	56 (18.1–77.5) [44.3–62.6]	46 (18–74.2) [34.7–59.3]	< 0.001
**Year allo-HSCT**			
Median (range) [IQR]	2014 (2010–2017)	2016 (2010–2017)	< 0.001
**Time diagnosis to allo-HSCT**			
Median (range) [IQR]	5.4 (1.5–17.7) [4.4–6.6]	4.7 (1.8–17.9) [3.8–7.7]	0.1
**AML**			
De novo	1206 (83.06%)	161 (92.53%)	0.001
secAML	246 (16.94%)	13 (7.47%)	
**Cytogenetics (MRC)**			
Good	59 (4.06%)	4 (2.3%)	0.19
Interm	740 (50.96%)	80 (45.98%)	
Poor	291 (20.04%)	35 (20.11%)	
NA/failed	362 (24.93%)	55 (31.61%)	
**Conditioning regimen**			
MAC	687 (47.31%)	76 (43.68%)	0.36
RIC	765 (52.69%)	98 (56.32%)	
**Gaft cell type**			
BM	143 (9.85%)	18 (10.34%)	0.84
PBSC	1309 (90.15%)	156 (89.66%)	
**Kanofsky performance score**			
< 90	331 (24.68%)	29 (16.76%)	0.02
≥ 90	1010 (75.32%)	144 (83.24%)	
Missing	111	1	
**Patient sex**			
Male	759 (52.27%)	98 (56.32%)	0.31
Female	693 (47.73%)	76 (43.68%)	
**Female donor-male recipient**	165 (11.41%)	26 (14.94%)	0.17
**Patient CMV serostatus**			
Negative	499 (34.92%)	43 (25.15%)	0.011
Positive	930 (65.08%)	128 (74.85%)	
Missing	23	3	
**Engraftement**			
Graft failure	12 (0.83%)	2 (1.16%)	0.65
Engrafted	1432 (99.17%)	170 (98.84%)	
Missing	8	2	

Abbreviations: *allo-HSCT* allogeneic stem cell transplantation, *AML* acute myeloid leukemia, *ATG* anti-thymocyte globulin, *BM* bone marrow, *CMV* cytomegalovirus, *Interm* intermediary, *IQR* interquartile range, *KPS* Karnovsky Performance Status, *MAC* myeloablative conditioning regimen, *PBSC* peripheral blood stem cell, *PTCY* posttransplantation cyclophosphamide, *RIC* reduced intensity conditioning regimen, *secAML* secondary acute myeloid leukemia

### Comparative analysis of transplant outcomes with ATG or PTCY

**Fig. 1:**
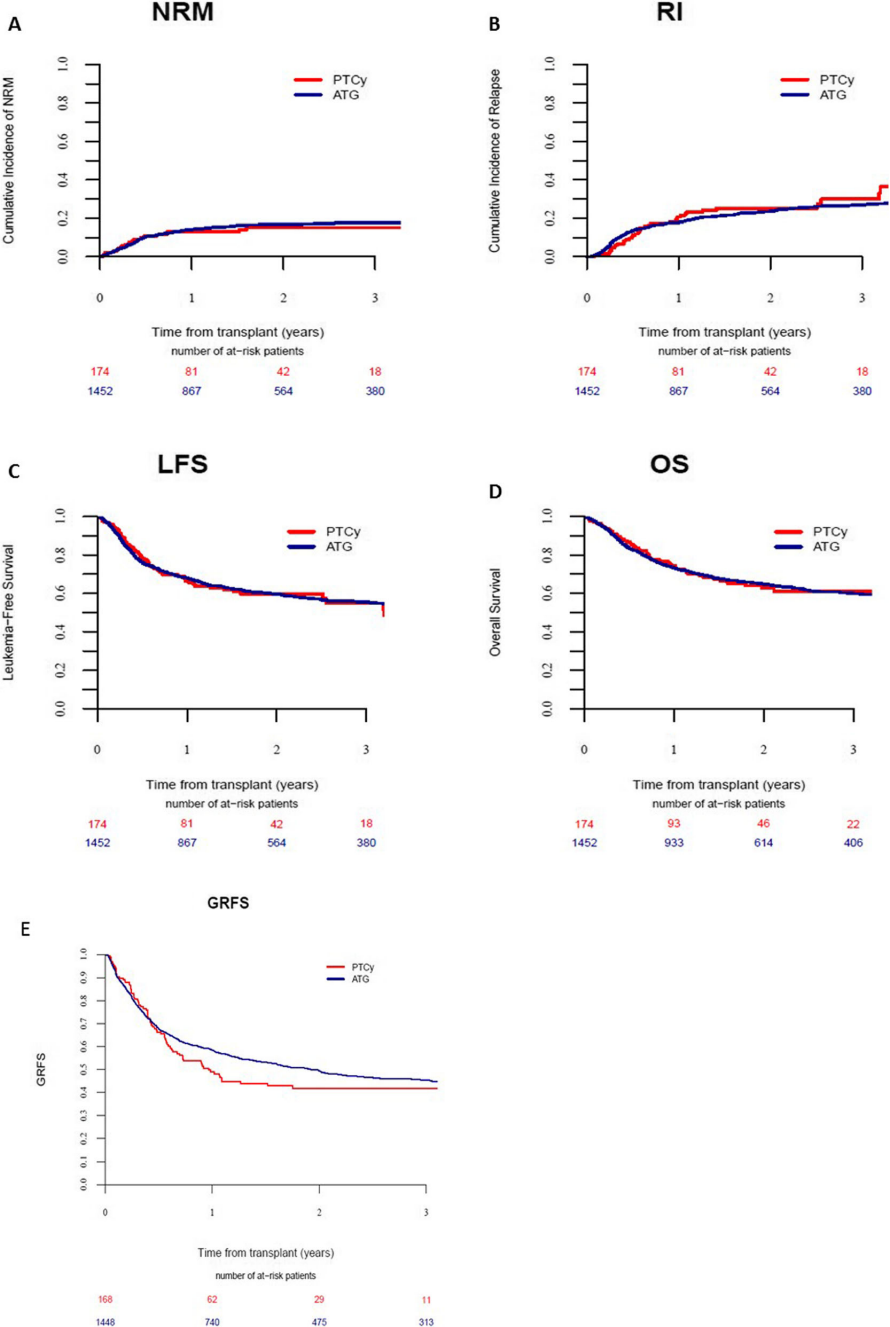
Non-relapse mortality (NRM) (**a**), relapse incidence (RI) (**b**), leukemia-free survival (LFS) (**c**), overall survival (OS) (**d**), graft-versus-host diseasefree, and relapse-free survival (GRFS) (**e**)

The results of uni- and multivariate analyses are summarized in Tables 2, 3, and 4. No statistically significant differences were observed between the PTCY and ATG groups for RI, NRM, LFS, OS, and GRFS (Fig. 1). These results were confirmed using matched-pair analysis (Tables S2 and S3).

**Table 2:** Cumulative incidence of GVHD

	180-day acute GVHD II–IV	180-day acute GVHD III–IV	2-year chronic GVHD	2-year ext. chronic GVHD
**PTCY**	28.8% [22.2–35.7]	8.8% [5.1–13.7]	31.4% [23.3–39.8]	18.5% [12–26.1]
**ATG**	29.2% [26.8–31.6]	9% [7.6–10.6]	33.6% [31–36.2]	13.1% [11.2–15]
***p*value**	0.68	0.89	0.43	0.11

Abbreviations: *ATG* antithymocyte globulin, *Ext* extensive, *GVHD* graft-versus host disease, *PTCY* post-transplantation cyclophosphamide

**Table 3:** Multivariate analysis for GVHD

	Acute GVHD II–IV		Acute GVHD III–IV		Chronic GVHD		Ext. chronic GVHD	
	HR (95% CI)	*p* value	HR (95% CI)	*p* value	HR (95% CI)	*p* value	HR (95% CI)	*p* value
ATG vs PTCY	0.98 (0.66–1.46)	0.93	0.84 (0.42–1.71)	0.64	1.22 (0.79–1.87)	0.37	0.64 (0.37–1.09)	0.09
Age (per 10 years)	1 (0.91–1.09)	0.92	1.01 (0.86–1.18)	0.92	1.06 (0.97–1.16)	0.20	1.03 (0.89–1.18)	0.73
sec. AML vs de novo	1.23 (0.95–1.61)	0.12	1.26 (0.79–2)	0.34	1.01 (0.76–1.33)	0.96	1.62 (1.1–2.39)	0.01
Adverse cytogenetics vs other	0.94 (0.74–1.21)	0.65	1.12 (0.73–1.71)	0.60	0.73 (0.56–0.96)	0.03	0.61 (0.39–0.95)	0.03
Female donor-male recipient vs other	1.27 (0.96–1.68)	0.10	1.67 (1.05–2.66)	0.03	1.07 (0.79–1.45)	0.65	1.05 (0.66–1.67)	0.83
RIC vs MAC	0.79 (0.62–1)	0.046	0.89 (0.59–1.36)	0.60	0.97 (0.76–1.23)	0.79	0.78 (0.54–1.14)	0.20
KPS ≥ 90	0.83 (0.65–1.06)	0.13	0.64 (0.42–0.97)	0.03	0.91 (0.71–1.17)	0.45	0.92 (0.63–1.34)	0.67
Patient CMV positivity	1.05 (0.85–1.31)	0.64	1.01 (0.68–1.48)	0.98	1.28 (1.03–1.6)	0.03	1.05 (0.75–1.47)	0.77
Donor CMV positivity	1 (0.81–1.23)	0.99	1.06 (0.73–1.53)	0.77	0.97 (0.79–1.2)	0.80	1.32 (0.96–1.82)	0.09
Year of transplantation	1.01 (0.96–1.06)	0.75	1.03 (0.94–1.12)	0.57	1 (0.95–1.05)	0.94	1.07 (0.99–1.16)	0.09
PBSC vs BM	1.05 (0.75–1.47)	0.77	1.1 (0.6–2)	0.76	1.1 (0.78–1.55)	0.59	1.37 (0.79–2.4)	0.26

Abbreviations: *aGVHD* acute graft-versus-host disease, *AML* acute myeloid leukemia, *ATG* antithymocyte globulin, *BM* bone marrow, *CI* confidence interval, *CMV* cytomegalovirus, *cGVHD* chronic graft-versus-host disease, *Ext* extensive, *HR* hazard ratio, *KPS* Karnofsky Performance Status, *MAC* myeloablative conditioning regimen, *PBSC* peripheral blood stem cells, *PTCY* post-transplantation cyclophosphamide, *RIC* reduced intensity conditioning regimen, *sec. AML* secondary acute myeloid leukemia

**Table 4:** Two-year survival outcomes

	Relapse	NRM	LFS	OS	GRFS
**PTCY**	25.2% [18–32.9]	15.2% [9.7–21.8]	59.7% [50.6–67.6]	62.7% [53.4–70.7]	41.6% [33–50]
**ATG**	23.7% [21.4–26]	16.7% [14.8–18.8]	59.6% [56.8–62.2]	64.8% [62.1–67.4]	49.3% [46.6–52.1]
***p*value**	0.6	0.6	0.97	0.95	0.2

Abbreviations: *ATG* antithymocyte globulin, *GRFS* GVHD-free, relapse-free survival, *LFS* leukemia-free survival, *OS* overall survival, *NRM* non-relapse mortality, *PTCY* posttransplantation cyclophosphamide

### Engraftment and GVHD

The proportion of patients achieving neutrophil engraftment at 100 days was similar in the PTCY and ATG groups (98.8% versus 99.2%, respectively, *p* = 0.65). The median time to neutrophil engraftment was 21 and 18 days in PTCY and ATG groups, *p* < 0.001.

**Fig. 2:**
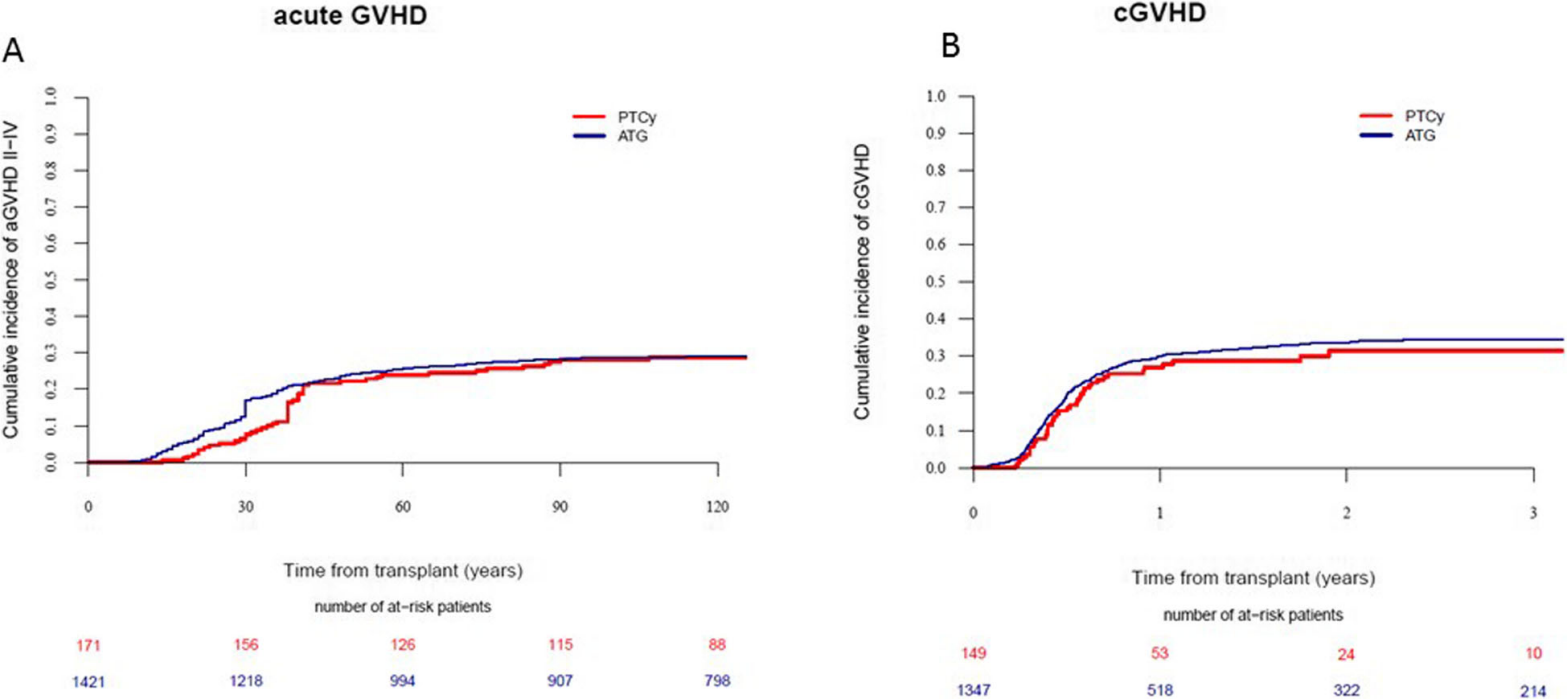
Cumulative incidence of GVHD. aGVHD (**a**) and cGVHD (**b**)

There were no significant differences in CI at 100 days of aGVHD (grades II–IV or III–IV), cGVHD, or ext cGVHD between the PTCY and the ATG group (Table 2) (Fig. 2). In the Cox model and in propensity score (data not shown), there was not a significant statistical difference between both groups, considering aGVHD II-IV, aGVHD III-IV, cGVH, and extensive cGVHD.

Regardless of the use of PTCY or ATG, a RIC regimen was independently associated with a lower risk of grade II–IV acute GVHD, female donor to male recipient and KPS < 90 associated with a higher risk of grade III–IV acute GVHD and patient CMV positivity with a higher risk of chronic GVHD (Table 3).

### OS, LFS, and GRFS

On univariate analysis, there were no significant differences between the two groups with respect to OS, LFS, or GRFS (Table 4). The GRFS was also similar, accounting for 42% in the PTCY and 49% in the ATG group (*p* = 0.2) which results were also confirmed in the multivariate analysis (Table 5). Regardless of the use of PTCY or ATG, a diagnosis of secondary AML and the presence of adverse cytogenetics were associated with lower probabilities of LFS, OS, and GRFS. Older age was also associated with a lower OS and LFS.

**Table 5:** Multivariate analysis for Relapse, NRM, LFS, OS, and GRFS

	Relapse		NRM		LFS		OS		GRFS	
	HR (95% CI)	*p* value	HR (95% CI)	*p* value	HR (95% CI)	*p* value	HR (95% CI)	*p* value	HR (95% CI)	*p* value
ATG vs PTCy	0.93 (0.63–1.37)	0.71	1.04 (0.65–1.66)	0.86	0.98 (0.74–1.29)	0.86	0.94 (0.7–1.27)	0.71	0.89 (0.7–1.13)	0.35
Age (per 10 years)	1.01 (0.92–1.11)	0.9	1.37 (1.2–1.56)	**< 10–5**	1.13 (1.05–1.22)	**0.001**	1.21 (1.12–1.32)	**< 10–5**	1.06 (1–1.14)	0.07
sec. AML vs de novo	1.14 (0.86–1.53)	0.36	1.39 (1.02–1.9)	**0.04**	1.24 (1.01–1.53)	**0.04**	1.29 (1.03–1.6)	**0.02**	1.25 (1.03–1.52)	**0.02**
Adverse cytogenetics vs other	1.77 (1.41–2.24)	**< 10–5**	1.19 (0.88–1.61)	0.27	1.51 (1.26–1.81)	**< 10–5**	1.42 (1.17–1.72)	**0.0004**	1.36 (1.15–1.61)	**0.0003**
Female donor-male recipient vs other	0.54 (0.36–0.8)	**0.002**	1.46 (1.04–2.05)	**0.03**	0.89 (0.69–1.15)	0.38	0.93 (0.71–1.22)	0.6	1.11 (0.89–1.38)	0.35
RIC vs MAC	1.06 (0.82–1.36)	0.66	0.84 (0.63–1.13)	0.25	0.97 (0.81–1.17)	0.75	0.91 (0.74–1.11)	0.33	0.98 (0.83–1.15)	0.77
KPS ≥ 90	1.16 (0.9–1.51)	0.26	0.85 (0.64–1.13)	0.27	1.02 (0.84–1.23)	0.86	0.98 (0.81–1.2)	0.87	0.96 (0.81–1.13)	0.60
Patient CMV positivity	0.99 (0.79–1.24)	0.92	1.31 (0.99–1.73)	0.06	1.11 (0.93–1.32)	0.24	1.12 (0.93–1.35)	0.22	1.11 (0.95–1.29)	0.21
Donor CMV positivity	1.17 (0.94–1.45)	0.16	1 (0.77–1.3)	0.98	1.09 (0.92–1.28)	0.33	1.09 (0.91–1.3)	0.34	1.11 (0.96–1.29)	0.16
Year of transplantation	1.04 (0.99–1.1)	0.12	1 (0.94–1.07)	0.99	1.02 (0.98–1.07)	0.26	1.02 (0.98–1.07)	0.37	1.03 (1–1.07)	0.07
PBSC vs BM	0.96 (0.68–1.36)	0.82	1.29 (0.8–2.09)	0.29	1.05 (0.8–1.39)	0.73	1.11 (0.82–1.49)	0.50	1.14 (0.88–1.47)	0.31

Abbreviations: *AML* acute myeloid leukemia, *ATG* antithymocyte globulin, *BM* bone marrow, *CI* confidence interval, *CMV* cytomegalovirus, *cGVHD* chronic graft-versus-host disease, *Ext* extensive, *GRFS* graft-versus-host disease-free, relapse-free survival, *HR* hazard ratio, *KPS* Karnofsky Performance Status, *LFS* leukemia-free survival, *MAC* myeloablative conditioning regimen, *NRM* non-relapse mortality, *OS* overall survival, *PBSC* peripheral blood stem cell, *PTCY* post-transplantation cyclophosphamide, *RIC* reduced intensity conditioning regimen, *sec.AML* secondary acute myeloid leukemia

### Relapse incidence and NRM

The 2-year RI and NRM rates did not differ between the two groups at 2 years. This was confirmed in the multivariate analysis where, regardless of the immunosuppressive agent used, adverse cytogenetics at diagnosis was independently associated with a higher risk of relapse. Female donor to male recipient transplants were associated with a lower risk of relapse. Older age, secondary AML patients, and transplants from a female donor to a male recipient were independently associated with a higher NRM (Table 5).

The main cause of death was disease recurrence in 47% of patients receiving PTCY and 39% of those receiving ATG. Infection accounted for 17% of deaths in the PTCY group and 22% of the ATG group. Cardiac toxicity was fatal for 1.9% of patients who received PTCY and 1.2% who received ATG (results not shown).

## Discussion

We have compared the impact of PTCY with that of ATG, (Thymoglobulin), in the conditioning regimen for patients undergoing transplantation from 10/10 MUD. First, we observed that PTCY and ATG had comparable cumulative incidences of aGVHD II–IV and grades III–IV with between 8 and 9% in each group. The impact was also similar considering cGVHD and extensive cGVHD. Considering ATG, these results were consistent with randomized clinical trials evaluating the use of ATG in HSCT from unrelated donors [7, 9, 10, 32, 33]. Most data about using PTCY in MUD are from studies of intensive pre-transplant conditioning regimens and mostly unmanipulated BM grafts. Luznik et al. reported data from 117 patients with high-risk hematological neoplasms transplanted from HLA-matched-related donors and MUDs after conditioning with busulfan and cyclophosphamide [34]. At 2 years after transplantation, the cumulative incidence of cGVHD for recipients of unrelated donor grafts was 11% (95% CI, 3–25%). It should be noted that PTCY was the only GVHD prophylaxis used, and BM was the only graft source used. Ruggeri et al. reported outcomes of 423 patients who received PTCY alone or in combination with additional drugs after HLA-matched sibling (*N* = 241) or MUD (*N* = 182) transplants using MAC or RIC [24]. In their study, 64% received PBSC. On multivariate analysis, PBSC was associated with a significantly higher risk of cGVHD and extensive cGVHD but had no impact on the other outcomes. We did not find any impact of source graft in our study; however, almost 90% of the patients received PBSC. Our results suggest that PTCY is an alternative to the recommended clinical practice of ATG in MUD. One hypothesis is that the degree of disparity between a recipient with a 10/10 HLA matched unrelated donor is low and the effect of PTCY of minimizing other HLA major or minor histocompatibility mismatches is not needed in this situation.

The second point is the absence of a significant difference at 2 years, in terms of NRM, which was quite low in both groups (16.7% and 15.2% for PTCY and ATG groups, respectively). Due to the retrospective nature of the study, we could not compare the CI of infections especially viral infections. Indeed, the use of ATG has been associated with EBV reactivation [9, 35]. The comparison between PTCY and ATG on EBV reactivation should be evaluated in prospective studies. In our study, however, the incidence of death from infection was similar in the two groups. Likewise, it would be of interest to report cardiac complications especially in the PTCY group, noting, that a similar incidence of death from cardiac failure was observed in both of our groups. RI was not statistically different in the two groups. Retrospective or non-randomized studies have reported conflicting results on the impact of ATG in the setting of RIC transplants. In particular, higher doses of ATG have been associated with a higher risk of relapse, thus leading to a decreased disease-free survival [11, 36]. On the contrary, Baron et al. found that the use of ATG was not significantly associated with a higher risk of relapse in patients with AML who underwent PBSC transplantation from HLA-identical siblings after RIC in CR1 [37]. Two other studies of our group did not find the impact of ATG on relapse even in high-risk AML [38, 39]. Because of lack of statistical power, we could not study the impact of PTCY or ATG with respect to the conditioning regimen intensity; however, we decided to include only patients who received a low dose of ATG (5 mg/kg in total), which has not been associated with a higher incidence of relapse [40, 41].

## Conclusions

The use of PTCY for GVHD prophylaxis resulted in similar outcomes to those seen with ATG for patients who underwent allogeneic stem cell transplantation for AML in CR1 with a 10/10 HLA-matched donor. The impact of the number, type, and schedule of the associated immunosuppressive agents needs further investigation. Due to the retrospective nature and the limitations of our analysis, including the schedule of ATG or PTCY and the lack of aforementioned data (e.g., infections, disease biology), our results need to be confirmed by prospective controlled studies. A precise knowledge of specific morbidity induced by each type of prophylaxis would be of great interest in clinical practice to aid the choice between PTCY or ATG when considering comorbidities and infection risk. Our results do, however, provide further proof that both ATG and PTCY are valid GVHD prophylactic strategies for transplants from 10/10 HLA-MUD.

## Supplementary information

**Additional file 1: Table S1.** Combination of immunosuppressive drugs. **Table S2.** Patient, disease, and transplant characteristics after matched-pair analysis. **Table S3.** Two-year survival outcomes and CI of GVHD after matched-pair analysis. EBMT participating centers

## Data Availability

EB, ML, AN, and MM had full access to all the data in the study (available upon data-specific request).

## Abbreviations

aGVHD: Acute graft-versus-host disease
allo-HSCT: Allogeneic stem cell transplantation.
AML: Acute myeloid leukemia
ATG: Anti-thymocyte globulin
BM: Bone marrow
cGVHD: Chronic graft-versus-host disease
CMV: Cytomegalovirus
CR: Complete remission
IR: Cumulative incidence of relapse
GRFS: Graft-versus-host disease-free, relapse-free survival
Interm: Intermediary
IQR: Interquartile range
KPS: Karnovsky Performance Status
LFS: Leukemia-free survival
MAC: Myeloablative conditioning regimen
MUD: Matched-unrelated donor
NRM: Non-relapse mortality
OS: Overall survival
PBSC: Peripheral blood stem cell
PTCY: Posttransplantation cyclophosphamide
RIC: Reduced intensity conditioning regimen
secAML: Secondary acute myeloid leukemia
