# Supplementary material for: Post-transplant cyclophosphamide versus antithymocyte globulin in patients with acute myeloid leukemia in first complete remission undergoing allogeneic stem cell transplantation from 10/10 HLA-matched unrelated donors
Source: J Hematol Oncol. 2020 Jul 3;13:87. doi: 10.1186/s13045-020-00923-0 (PMC7333262; doi:10.1186/s13045-020-00923-0)
Supplement: Supplementary file 1 — Additional file 1: Table S1. Combination of immunosuppressive drugs. Table S2. Patient, disease, and transplant characteristics after matched-pair analysis. Table S3. Two-year survival outcomes and CI of GVHD after matched-pair analysis. EBMT participating centers [file 13045_2020_923_MOESM1_ESM.docx]

Supplementary Data

| Table S1. Combination of immunosuppressive drugs | | |  |  |
| --- | --- | --- | --- | --- |
|  |  | **ATG** | **PTCY** |  |
|  | csa | 201 (13.84%) | 57 (32.76%) |  |
|  | mtx | 13 (0.9%) | 11 (6.32%) |  |
|  | mmf | 1 (0.07%) | 0 (0%) |  |
|  | siro | 2 (0.14%) | 0 (0%) |  |
|  | tacro | 6 (0.41%) | 11 (6.32%) |  |
|  | csa+mtx | 655 (45.11%) | 5 (2.87%) |  |
|  | csa+mmf | 427 (29.41%) | 12 (6.9%) |  |
|  | csa+tacro | 6 (0.41%) | 0 (0%) |  |
|  | mtx+mmf | 8 (0.55%) | 0 (0%) |  |
|  | mmf+tacro | 47 (3.24%) | 57 (32.76%) |  |
|  | tacro+siro | 14 (0.96%) | 0 (0%) |  |
|  | mmf+siro | 2 (0.14%) | 11 (6.32%) |  |
|  | mtx+tacro | 51 (3.51%) | 0 (0%) |  |
|  | csa+mtx+mmf | 19 (1.31%) | 0 (0%) |  |
|  | other | 0 (0%) | 10 (5.75%) |  |
|  | abbreviations: ATG: antithymocyte globulin; PTCY: post-transplant cyclophosphamide; CsA: cyclosporin A; mtx: methotrexate; mmf: mycophenolate mofetil; siro: sirolimus; tacro: tacrolimus | | |  |
|  |  |  |  |  |
|  |  |  |  |  |
|  |  |  |  |  |
|  |  |  |  |  |

| **Table S2. Patient, disease, and transplant characteristics after matched-pair analysis** | | | | |  |
| --- | --- | --- | --- | --- | --- |
|  |  | **ATG** | **PTCy** | **Test p-value** |  |
| **N** |  | **246** | **125** |  |  |
| **Follow-up** | Median time (IQR) mo | 24.4 (15.4-39.3) | 31.0 (17.3-55.6) |  |  |
| **Patient age** | Median (range) (IQR) y | 51.5 (18.4-77.5) [ 40.4-61.3 ] | 49.6 (18.2-70.8) [ 38.8-62.7 ] | 0.71 |  |
| **Year of allo-HSCT** | Median (range) | 2015 (2010-2017) | 2016 (2010-2017) | < 0.001 |  |
| **Time from diagnosis to SCT** | Median time (range) (IQR) mo | 5.3 (1.5-16.9) [ 4.3-6.7 ] | 4.4 (1.8-17.9) [ 3.5-6.3 ] | < 0.001 |  |
| **AML characteristics** | de novo | 220 (89.43%) | 114 (91.2%) | 0.59 |  |
|  | secondary | 26 (10.57%) | 11 (8.8%) |  |  |
| **Cytogenetics** | good | 6 (2.44%) | 3 (2.4%) | 0.96 |  |
|  | interm | 112 (45.53%) | 59 (47.2%) |  |  |
|  | poor | 62 (25.2%) | 33 (26.4%) |  |  |
|  | NA/failed | 66 (26.83%) | 30 (24%) |  |  |
| **Conditioning regimen** | MAC | 106 (43.09%) | 54 (43.2%) | 0.98 |  |
|  | RIC | 140 (56.91%) | 71 (56.8%) |  |  |
| **Female to male combination** | no F->M | 207 (84.15%) | 106 (84.8%) | 0.87 |  |
|  | F->M | 39 (15.85%) | 19 (15.2%) |  |  |
| **Gaft cell type** | BM | 18 (7.32%) | 12 (9.6%) | 0.45 |  |
|  | PBSC | 228 (92.68%) | 113 (90.4%) |  |  |
| **Patient CMV** | Pat. CMV neg. | 65 (26.42%) | 37 (29.6%) | 0.52 |  |
|  | Pat. CMV pos | 181 (73.58%) | 88 (70.4%) |  |  |
| **Kanofsky performance score** | <90 | 43 (17.48%) | 22 (17.6%) | 0.98 |  |
|  | >=90 | 203 (82.52%) | 103 (82.4%) |  |  |
| **Donor CMV** | Don. CMV neg. | 126 (51.22%) | 66 (52.8%) | 0.77 |  |
|  | Don. CMV pos | 120 (48.78%) | 59 (47.2%) |  |  |
| Abbreviations: allo-HSCT= allogeneic stem cell transplantation. AML= acute myeloid leukemia. ATG= anti-thymocyte globulin. BM=bone marrow. CMV= cytomegalovirus. F=Female. Interm: intermediary. IQR=interquartile range. KPS=Karnovsky Performance Status. M= Male. MAC=myeloablative conditioning regimen. Mo= month. PBSC=peripheral blood stem cell. PTCY= posttransplantation cyclophosphamide. RIC=reduced intensity conditioning regimen. secAML= secondary acute myeloid leukemia. Y=year | | | | |  |
|  |  |  |  |  |  |
|  |  |  |  |  |  |
|  |  |  |  |  |  |

| **Table S3. Two-year survival outcomes and CI of GVHD after matched-pair analysis** | | | | |  |
| --- | --- | --- | --- | --- | --- |
| GVHD prophylaxis | **PTCY** | **ATG** | **HR (95% CI) p (stratified)** | **p (stratified)** |  |
| RI | 28.3%[20.1-37] | 22.9%[17.5-28.7] | 0.85 (0.52-1.40) | p=0.52 |  |
| NRM | 13.4%[7.8-20.6] | 15.9%[11.5-20.9] | 1.41 (0.74-2.70) | p=0.30 |  |
| LFS | 58.3%[48.3-67] | 61.2%[54.4-67.3] | 1.03 (0.70-1.53) | p=0.87 |  |
| OS | 62.1%[51.8-70.8] | 66.9%[60.2-72.8] | 1.12 (0.73-1.71) | p=0.60 |  |
| GRFS | 39.9%[30.5-49.1] | 51.9%[45.1-58.2] | 0.87 (0.62-1.23) | p=0.45 |  |
| Acute GVHD II-IV | 28.1%[20.5-36.2] | 27.3%[21.9-33] | 0.98 (0.64-1.49) | p=0.91 |  |
| Acute GVHD III-IV | 7.3%[3.6-12.7] | 8.1%[5.1-12] | (0.53-2.61) | p=0.69 |  |
| chronic GVHD | 30.7%[22.1-39.8] | 28.7%[23-34.8] | 0.95 (0.58-1.54) | p=0.84 |  |
| Ext. chronic GVHD | 19.5%[12.6-27.6] | 10.4%[6.9-14.8] | 0.56 (0.29-1.11) | p=0.09 |  |
| Abbreviations: ATG= antithymocyte globulin. Ext=extensive. GRFS= GVHD-free, relapse-free survival. GVHD= graft-versus-host disease. LFS= leukemia-free survival. OS= overall survival. NRM= non-relapse mortality. PTCY= posttransplantation cyclophosphamide. RI= relapse incidence | | | | |  |
|  |  |  |  |  |  |

| **EBMT participating centers** |  |  |  |  |  |
| --- | --- | --- | --- | --- | --- |
|  |  |  |  |  |  |
| **Entire population** | **Patients number N** | **Posttransplantation cyclophosphamaide** | **Patients number N** | **Antithymocyte globulin** | **Patients number N** |
| 644 Vilnius [Santariskiy Kl] | 91 | 725 St._Petersburg [Pavlov Med Univ] | 56 | 644 Vilnius [Santariskiy Kl] | 91 |
| 230 Marseille [Paoli Calmettes] | 73 | 246 Rotterdam [Erasmus MC] | 22 | 230 Marseille [Paoli Calmettes] | 72 |
| 267 Pessac [H Haut-Leveque] | 63 | 588 Amsterdam [VU Univ Med Ctr] | 19 | 267 Pessac [H Haut-Leveque] | 61 |
| 725 St._Petersburg [Pavlov Med Univ] | 56 | 565 Maastricht [Univ H] | 10 | 610 Bratislava [Univ H] | 44 |
| 212 Stockholm [Univ H] | 44 | 214 Barcelona [H Clinic] | 9 | 212 Stockholm [Univ H] | 42 |
| 610 Bratislava [Univ H] | 44 | 813 Milano [S Raffaele] | 7 | 270 Grenoble [H A Michallon] | 42 |
| 270 Grenoble [H A Michallon] | 43 | 819 Madrid [H G Marañón] | 4 | 515 Helsinki [Univ Central H] | 42 |
| 515 Helsinki [Univ Central H] | 42 | 546 Groningen [Univ H] | 3 | 233 Besancon [H Jean Minjoz] | 41 |
| 233 Besancon [H Jean Minjoz] | 41 | 614 Hamburg [Univ H] | 3 | 266 Uppsala [Univ H] | 37 |
| 266 Uppsala [Univ H] | 38 | 705 Udine [Univ H] | 3 | 671 Lyon [H E Herriot] | 35 |
| 209 Leuven [Univ H] | 35 | 209 Leuven [Univ H] | 2 | 209 Leuven [Univ H] | 33 |
| 671 Lyon [H E Herriot] | 35 | 212 Stockholm [Univ H] | 2 | 225 Turku [University] | 33 |
| 225 Turku [University] | 33 | 235 Oslo [Rikshospitalet] | 2 | 624 Toulouse [H Purpan] | 33 |
| 624 Toulouse [H Purpan] | 33 | 242 Santander [Valdecilla] | 2 | 926 Montpellier [University] | 33 |
| 926 Montpellier [University] | 33 | 267 Pessac [H Haut-Leveque] | 2 | 253 Nantes [Hotel Dieu] | 32 |
| 253 Nantes [Hotel Dieu] | 32 | 440 Kocaeli [Anadolu] | 2 | 659 Brest [C.H.R.U Brest] | 31 |
| 659 Brest [C.H.R.U Brest] | 31 | 663 Valencia [H Univ La Fe] | 2 | 289 Goeteborg [Sahlgrenska Univ H] | 29 |
| 289 Goeteborg [Sahlgrenska Univ H] | 29 | 230 Marseille [Paoli Calmettes] | 1 | 661 Rennes [H Sud/Pontchaillou] | 28 |
| 661 Rennes [H Sud/Pontchaillou] | 28 | 252 Creteil [H Mondor Hematol] | 1 | 387 Birmingham [Queen Elizabeth] | 27 |
| 387 Birmingham [Queen Elizabeth] | 27 | 261 Geneva [261] | 1 | 650 Angers [CHRU] | 26 |
| 650 Angers [CHRU] | 26 | 266 Uppsala [Univ H] | 1 | 523 Nice [H de l`ARCHET I] | 25 |
| 523 Nice [H de l`ARCHET I] | 25 | 270 Grenoble [H A Michallon] | 1 | 283 Lund [Univ H] | 24 |
| 283 Lund [Univ H] | 24 | 282 Valencia [H Clinico] | 1 | 977 Limoges [CHRU] | 24 |
| 977 Limoges [CHRU] | 24 | 284 Birmingham [Heartlands H] | 1 | 658 Bergamo [Ospedale, ematol] | 23 |
| 658 Bergamo [Ospedale, ematol] | 23 | 286 Pavia [S Matteo] | 1 | 251 Caen [Hopital, Hematol] | 21 |
| 246 Rotterdam [Erasmus MC] | 22 | 287 Rome [S C-Forlanini] | 1 | 389 Leipzig [Univ, Haemat/Oncol] | 21 |
| 251 Caen [Hopital, Hematol] | 21 | 304 Firenze [Careggi-Meyer] | 1 | 277 Lille [H Claude Huriez] | 20 |
| 389 Leipzig [Univ, Haemat/Oncol] | 21 | 338 Halle [Univ Martin-Luther] | 1 | 731 Umeå [Univ H] | 20 |
| 277 Lille [H Claude Huriez] | 20 | 409 Petach-Tikva [Beilinson H] | 1 | 746 Tartu [Univ H] | 20 |
| 731 Umeå [Univ H] | 20 | 427 Bucharest [Fundeni Clin Inst] | 1 | 264 Poitiers [H La Miletrie] | 19 |
| 746 Tartu [Univ H] | 20 | 428 Gliwice [Sklodowska] | 1 | 672 Strasbourg [H Hautepierre] | 19 |
| 264 Poitiers [H La Miletrie] | 19 | 566 Cambridge [Addenbrookes H] | 1 | 676 Vandoeuvre_Les_Nancy [Hosp] | 19 |
| 588 Amsterdam [VU Univ Med Ctr] | 19 | 584 Barcelona [V d`Hebron Adults] | 1 | 729 Hradec_Králové [Charles U H, Hem] | 18 |
| 672 Strasbourg [H Hautepierre] | 19 | 645 Marburg [Philipps Univ] | 1 | 207 Paris [St Louis] | 16 |
| 676 Vandoeuvre_Les_Nancy [Hosp] | 19 | 710 Perth [Royal H] | 1 | 666 Villejuif [Gustave Roussy] | 16 |
| 729 Hradec_Králové [Charles U H, Hem] | 18 | 763 London [Kings College H] | 1 | 160 Paris [H Necker] | 15 |
| 207 Paris [St Louis] | 16 | 766 Napoli [Federico II] | 1 | 693 Warsaw [Inst Haematology] | 15 |
| 666 Villejuif [Gustave Roussy] | 16 | 775 Paris [St Antoine] | 1 | 941 Rouen [Becquerel] | 15 |
| 160 Paris [H Necker] | 15 | 780 Manchester [Christie] | 1 | 262 Paris [Pitie-Salpetriere] | 14 |
| 693 Warsaw [Inst Haematology] | 15 | 792 Catania [Osp Ferrarotto] | 1 | 215 Brussels [Jules Bordet] | 13 |
| 941 Rouen [Becquerel] | 15 | 794 Perugia [Monteluce] | 1 | 273 Clermont-Ferrand [Jean Perrin] | 13 |
| 262 Paris [Pitie-Salpetriere] | 14 | Total | 174 | 252 Creteil [H Mondor Hematol] | 11 |
| 215 Brussels [Jules Bordet] | 13 |  |  | 775 Paris [St Antoine] | 11 |
| 273 Clermont-Ferrand [Jean Perrin] | 13 |  |  | 996 Antwerp_Edegem [UZA] | 11 |
| 252 Creteil [H Mondor Hematol] | 12 |  |  | 726 Liege [University] | 10 |
| 775 Paris [St Antoine] | 12 |  |  | 955 Amiens [H Sud] | 10 |
| 235 Oslo [Rikshospitalet] | 11 |  |  | 235 Oslo [Rikshospitalet] | 9 |
| 813 Milano [S Raffaele] | 11 |  |  | 248 Pescara [Osp Civile] | 9 |
| 996 Antwerp_Edegem [UZA] | 11 |  |  | 712 Wuerzburg [Medizinische Kl II] | 9 |
| 565 Maastricht [Univ H] | 10 |  |  | 526 San_Giovanni_Rotondo [IRCCS] | 7 |
| 726 Liege [University] | 10 |  |  | 598 San_Sebastian [H Aranzazu] | 7 |
| 955 Amiens [H Sud] | 10 |  |  | 744 Gent [Univ H] | 7 |
| 214 Barcelona [H Clinic] | 9 |  |  | 211 Sao_Paulo [H Sirio-Libanes] | 6 |
| 248 Pescara [Osp Civile] | 9 |  |  | 302 Zagreb [Univ H Rebro] | 6 |
| 712 Wuerzburg [Medizinische Kl II] | 9 |  |  | 428 Gliwice [Sklodowska] | 6 |
| 428 Gliwice [Sklodowska] | 7 |  |  | 566 Cambridge [Addenbrookes H] | 6 |
| 526 San_Giovanni_Rotondo [IRCCS] | 7 |  |  | 699 Wroclaw [Medical Acad] | 6 |
| 566 Cambridge [Addenbrookes H] | 7 |  |  | 811 Cagliari [Osp Businco] | 6 |
| 598 San_Sebastian [H Aranzazu] | 7 |  |  | 339 Antwerp [AZ Stuivenberg] | 5 |
| 744 Gent [Univ H] | 7 |  |  | 601 Manchester [Royal Infirmary] | 5 |
| 211 Sao_Paulo [H Sirio-Libanes] | 6 |  |  | 238 Córdoba [Reina Sofia] | 4 |
| 302 Zagreb [Univ H Rebro] | 6 |  |  | 456 Pretoria [Albert Albert] | 4 |
| 614 Hamburg [Univ H] | 6 |  |  | 574 Olomouc [Univ H] | 4 |
| 699 Wroclaw [Medical Acad] | 6 |  |  | 717 Nottingham [City H] | 4 |
| 811 Cagliari [Osp Businco] | 6 |  |  | 763 London [Kings College H] | 4 |
| 339 Antwerp [AZ Stuivenberg] | 5 |  |  | 785 Homburg [Univ Saarland] | 4 |
| 601 Manchester [Royal Infirmary] | 5 |  |  | 813 Milano [S Raffaele] | 4 |
| 763 London [Kings College H] | 5 |  |  | 234 Brussels [St. Luc] | 3 |
| 238 Córdoba [Reina Sofia] | 4 |  |  | 254 Leeds [St James] | 3 |
| 456 Pretoria [Albert Albert] | 4 |  |  | 255 Oxford [Radcliffe H] | 3 |
| 574 Olomouc [Univ H] | 4 |  |  | 300 Lisboa [Inst Oncologia] | 3 |
| 663 Valencia [H Univ La Fe] | 4 |  |  | 345 Haifa [Rambam MCH] | 3 |
| 717 Nottingham [City H] | 4 |  |  | 386 Bristol [Royal H Sick Chil] | 3 |
| 785 Homburg [Univ Saarland] | 4 |  |  | 397 Riyadh [King Faisal] | 3 |
| 819 Madrid [H G Marañón] | 4 |  |  | 614 Hamburg [Univ H] | 3 |
| 234 Brussels [St. Luc] | 3 |  |  | 615 Madrid [H Ramón y Cajal] | 3 |
| 254 Leeds [St James] | 3 |  |  | 740 Linköping [Univ H] | 3 |
| 255 Oxford [Radcliffe H] | 3 |  |  | 788 Ancona [Umberto I] | 3 |
| 261 Geneva [261] | 3 |  |  | 970 Flensburg [St Franziskus] | 3 |
| 300 Lisboa [Inst Oncologia] | 3 |  |  | 244 Glasgow [Royal Infirmary] | 2 |
| 345 Haifa [Rambam MCH] | 3 |  |  | 261 Geneva [261] | 2 |
| 386 Bristol [Royal H Sick Chil] | 3 |  |  | 534 Cologne [Univ, Medicine] | 2 |
| 397 Riyadh [King Faisal] | 3 |  |  | 561 Thessaloniki [Papanicolaou G H] | 2 |
| 546 Groningen [Univ H] | 3 |  |  | 570 Santiago_De_Compostela [H Clin Univ] | 2 |
| 615 Madrid [H Ramón y Cajal] | 3 |  |  | 606 Cuneo [S Croce e Carle] | 2 |
| 705 Udine [Univ H] | 3 |  |  | 633 Teheran [Shariati] | 2 |
| 740 Linköping [Univ H] | 3 |  |  | 663 Valencia [H Univ La Fe] | 2 |
| 788 Ancona [Umberto I] | 3 |  |  | 713 Leicester [Royal Infirmary] | 2 |
| 970 Flensburg [St Franziskus] | 3 |  |  | 797 Vicenza [Osp S Bartolo] | 2 |
| 242 Santander [Valdecilla] | 2 |  |  | 208 Zürich [208] | 1 |
| 244 Glasgow [Royal Infirmary] | 2 |  |  | 232 Rome [Emat, La Sapienza] | 1 |
| 286 Pavia [S Matteo] | 2 |  |  | 239 Utrecht [University] | 1 |
| 304 Firenze [Careggi-Meyer] | 2 |  |  | 286 Pavia [S Matteo] | 1 |
| 409 Petach-Tikva [Beilinson H] | 2 |  |  | 304 Firenze [Careggi-Meyer] | 1 |
| 440 Kocaeli [Anadolu] | 2 |  |  | 307 Rome [Univ S Cuore] | 1 |
| 534 Cologne [Univ, Medicine] | 2 |  |  | 308 Graz [Medical Univ] | 1 |
| 561 Thessaloniki [Papanicolaou G H] | 2 |  |  | 356 Salzburg [LKA, Oncology] | 1 |
| 570 Santiago_De_Compostela [H Clin Univ] | 2 |  |  | 409 Petach-Tikva [Beilinson H] | 1 |
| 606 Cuneo [S Croce e Carle] | 2 |  |  | 445 Istanbul [Medipol Adult] | 1 |
| 633 Teheran [Shariati] | 2 |  |  | 533 Jena [Friedrich-Schiller] | 1 |
| 710 Perth [Royal H] | 2 |  |  | 539 London [St George`s] | 1 |
| 713 Leicester [Royal Infirmary] | 2 |  |  | 552 Goettingen [Univ Kl] | 1 |
| 794 Perugia [Monteluce] | 2 |  |  | 556 Budapest [National Med Ctr] | 1 |
| 797 Vicenza [Osp S Bartolo] | 2 |  |  | 622 Athens [Evangelismos H] | 1 |
| 208 Zürich [208] | 1 |  |  | 656 Prague [Ist Hematology] | 1 |
| 232 Rome [Emat, La Sapienza] | 1 |  |  | 710 Perth [Royal H] | 1 |
| 239 Utrecht [University] | 1 |  |  | 778 Sheffield [Royal Hallamshire] | 1 |
| 282 Valencia [H Clinico] | 1 |  |  | 794 Perugia [Monteluce] | 1 |
| 284 Birmingham [Heartlands H] | 1 |  |  | Total | 1452 |
| 287 Rome [S C-Forlanini] | 1 |  |  |  |  |
| 307 Rome [Univ S Cuore] | 1 |  |  |  |  |
| 308 Graz [Medical Univ] | 1 |  |  |  |  |
| 338 Halle [Univ Martin-Luther] | 1 |  |  |  |  |
| 356 Salzburg [LKA, Oncology] | 1 |  |  |  |  |
| 427 Bucharest [Fundeni Clin Inst] | 1 |  |  |  |  |
| 445 Istanbul [Medipol Adult] | 1 |  |  |  |  |
| 533 Jena [Friedrich-Schiller] | 1 |  |  |  |  |
| 539 London [St George`s] | 1 |  |  |  |  |
| 552 Goettingen [Univ Kl] | 1 |  |  |  |  |
| 556 Budapest [National Med Ctr] | 1 |  |  |  |  |
| 584 Barcelona [V d`Hebron Adults] | 1 |  |  |  |  |
| 622 Athens [Evangelismos H] | 1 |  |  |  |  |
| 645 Marburg [Philipps Univ] | 1 |  |  |  |  |
| 656 Prague [Ist Hematology] | 1 |  |  |  |  |
| 766 Napoli [Federico II] | 1 |  |  |  |  |
| 778 Sheffield [Royal Hallamshire] | 1 |  |  |  |  |
| 780 Manchester [Christie] | 1 |  |  |  |  |
| 792 Catania [Osp Ferrarotto] | 1 |  |  |  |  |
| Total | 1626 |  |  |  |  |
